# Effect of self-instructional module towards the prevention of cataract among elderly people in India

**DOI:** 10.6026/973206300191051

**Published:** 2023-11-30

**Authors:** Nisha K, Ugin Juliyet MP, Sadhana Undru, Swapna Dileep, Swapna Kumari Andugula

**Affiliations:** 1 Department of Community Health Nursing, KIMS Nursing College, KIMS & RF Amalapuram, East Gothwari - 533201, Andhra Pradesh, India; 2 Department of Community Health Nursing, Bon Secours Nursing College, Molachur, 602106, Tamil Nadu; 3 Department of Psychiatric Nursing, KIMS Nursing College, KIMS & RF Amalapuram, East Gothwari -533201, Andhra Pradesh, India; 4 Department of Obstructive Gynecology Nursing, KIMS Nursing College, KIMS & RF Amalapuram, East Gothwari - 533201, Andhra Pradesh, India

**Keywords:** Cataract, self-instructional module, knowledge of cataract

## Abstract

The leading cause of blindness in the world is cataracts, which are dangerous and curable with proper eye care. Eye care service is thought to play significant role in prevention. Nonetheless, not much research has been done to gauge older persons' in rural India's level of cataract awareness and how it relates to their use of eye care services. Therefore, in order to prevent cataracts in the elderly, we described a self-instruction model for cataract knowledge and looked into the variations between pre- and post-test self-instruction models. Their demographic characteristics showed that the higher age group female had highest prevalence of cataracts. The study population's understanding of cataracts is incredibly low: only 2% of participants have adequate knowledge, 52% have somewhat adequate information, and 46% have inadequate knowledge. However, after completing the self-instructional module, 54% of participants felt they knew enough to prevent cataracts. The results of the study showed that the self-instructional module was very helpful in helping the senior population learn about cataract prevention. More interventional research with a larger sample size should be carried out to gain a better understanding of cataract prevention in older adults,.

## Background:

A cataract is described as a decrease in lens transparency brought on by lens opacification [1]. Cataracts can be categorized into age-related, paediatric, and other causes of cataracts, depending on their underlying causes [2]. Vision loss and ultimate blindness could result from this haziness of the lens. According to the World Health Organization (WHO), 65.2 million people are suffering from moderate or severe cataract; about 18 million people worldwide are estimated to be bilaterally blind from cataracts, accounting for nearly half of all blindness cases worldwide [3]. Around the world, cataracts continue to be the principal cause of blindness and a significant factor in visual impairment [4]. Women are less likely than men to follow the World Health Organization's 2014-2019 WHO Action Plan for the Prevention of Blindness (WHA66/11), and they also have a higher risk of cataracts; Gathering in May of 2013 metrics for improving global programme monitoring, data collecting, and service delivery metrics pertinent to cataracts are all included in the action plan. [5]. The population is growing, especially among the elderly, thus it is anticipated that the number will continue to grow [6]. The ICMR Survey 2017 indicates that the prevalence of cataracts has increased due to the growing older population brought on by improved healthcare and other factors [7]. The removal of the cataract and implantation of an intraocular lens (IOL) during cataract surgery allows for immediate vision recovery [8]. Despite the ease with which cataracts can be treated and the fact that cataract surgery is among the most cost-effective procedures, many isolated and underdeveloped regions of the developing world continue to see a high rate of cataract-related blindness, largely because of limited access to eye care [9]. According to the International Agency for the Prevention of Blindness, the percentage of blindness caused by cataracts among all eye illnesses varies from 5% in wealthy nations to 50% or more in impoverished and/or isolated areas [10].

In accordance with the five-year national Strategic Vision 2000 plans, district-level Vision 2020 programmes are created as a one-year operational plan and established as important parts of the district general health operational plan [11]. Every district vision 2020 plan should, to the greatest extent possible, be horizontal, integrated into the current district health service structure, and consistent with primary health care principles [12]. These guidelines include equity, community involvement, an emphasis on technology that is suited for prevention, and a multi-sectoral strategy [13].

District programmes offer a continuum of thorough eye care, including promotion of eye health, disease prevention, curative intervention, and rehabilitation [14]. These four components have hitherto operated separately and without coordination [15]. The four components have been coordinated and given attention. Eye care that is comprehensive should be accessible, inexpensive, and responsible [16]. Even with all these advancements, the majority of people living in rural areas still cannot afford quality eye care [10]. Up until recently, surgical and screening eye camps have been the core of eye care services in the majority of these communities, and it has long been debatable how well these camps deliver their services [17]. It is essential to build such facilities at the district level in order to consistently and permanently deliver high-quality service to the population in rural and suburban areas [18]. The worldwide campaign to combat the issue of preventing blindness by the year 2020 suggested that there should be a secondary-level eye care facility for every 100,000 people [19]. In 2003, at the 56th World Health Assembly, our ministry of health pledged its support for Vision 2020 [20]. With assistance from global and neighbourhood non-governmental development organisations (NGDO), they ought to be the main proprietors of district-level Vision 2020 projects [21]. The objective of the current study was to assess the efficacy of information on the control and prevention of cataracts among senior people, despite the fact that the government and programmes can address cataract-related blindness. Study the level of knowledge prior to and following the self-instruction module, and focus on the self-instructional module of knowledge as a preventative and control the cataract.

## Methodology:

## Research design:

**Figure 1:**
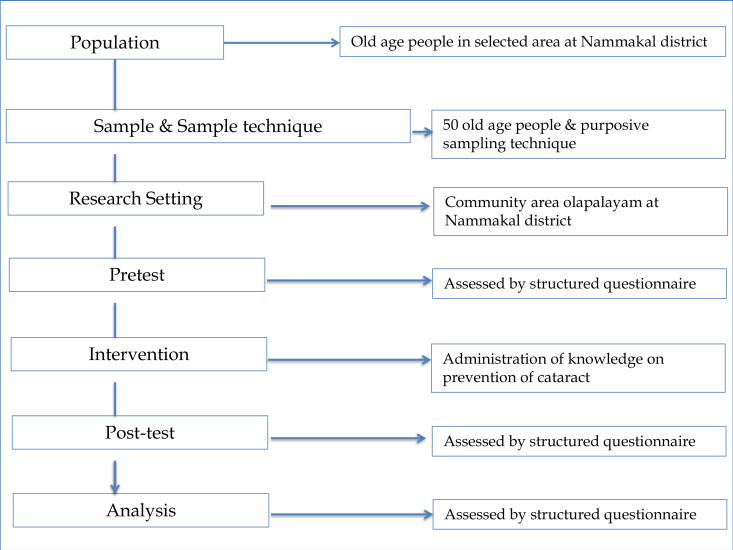
Research design for evaluate self-instructional module for the prevention of cataracts among elderly people

Qualitative descriptive method was chosen to assess the knowledge on prevention and management on senile cataract among older adults (Figure 1 and Table 1).

## Study design:

A quantitative-evaluative approach is adopted for the present study Table 2. A pre-experimental group pre-test and post-experimental group post-test design was selected for this study to attain knowledge on the prevention and management of cataracts among older adults. The sample size in the current study was determined according to the following formula for comparing means between two independent groups: Considering a type one error rate of 5%, statistical power of 80% for detecting standardised effect size (Δ = 0.8) for study main outcomes, and an equal number of patients in each group (ϕ = 1), this leads to 50 samples.

The study design is depicted as shown below,

## Setting of the study:

The study was carried out in Tamil Nadu's Olapalayam, Namakkal district. The self-instructional module must be completed in a setting that is conducive, peaceful, and distraction-free. The complete membership of the group in which the researcher was interested in extrapolating the study's findings is included in the target population. The elderly were the study's target population.

## Ethnical consideration:

Has got ethical clearance being obtained from the Olapalayam village leader

## Data collection:

The data collection instrument comprises of two parts. Part-I consists of demographic variables and part- II incorporates the questionnaire on knowledge.

## Part I:

This section encloses information regarding demographic variables such as age, religion, educational status, type of employment, marital status, health status, monthly income and source of information for the peoples.

## Part II:

This section consists of questionnaire of 30 questions to assess the knowledge of peoples regarding cataract. A score of 1 is given for each correct response and a score of 0 is given for incorrect response.

## Data collecting timeframe:

The primary study was carried out in Olapalayam between February 2 and March 2, 2021. The chief of town gave the investigator permission. A structured questionnaire was used to gather the information. For the investigation, 50 samples were chosen. The samples were informed of the study's goal. A systematic questionnaire with 10 items was used to gather and evaluate a demographic profile.

## Plan for data analysis:

Statistics that are both descriptive and inferential were used to analyze the data. The chi square test was used to determine the connection.

## Results:

Analysis and interpretation of the information gathered to gauge people's cataract knowledge Inferential and descriptive statistics that were appropriate were used to evaluate and interpret the data.

## Section-A: Description of samples according to their demographic variables.

Table 3 shows that, out of 50 samples, women had the highest frequency of cataracts (60%) in the 65-75 age range. Religion-wise, Hinduism is more common (80%). According to their marital status, 88% of them were married at a high frequency. As far as they know, 28% of them had a high frequency of cataracts in elementary school. Government workers were more likely to have cataracts (36%), based on their line of work, non-vegetarians (68%), in accordance with their eating habits, were more likely to develop cataracts; of those who received treatment, 40% did so in health centres. Furthermore, their family type indicated that 56% of them were members of nuclear households and that cataract was more common in the elderly.

## Section B: Assess the level of knowledge of peoples gained knowledge from self-instructional module on control and prevention of cataract.

**Figure 2:**
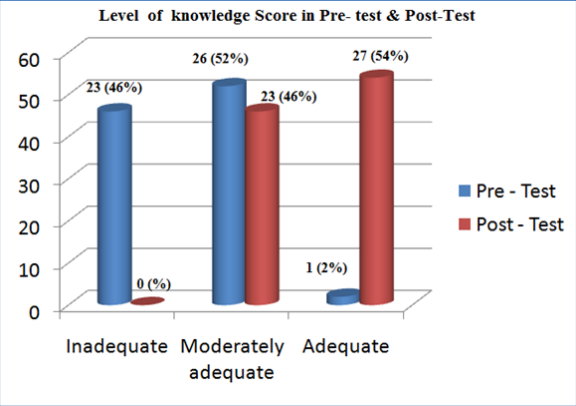
Frequency and percentage distribution of pre and post-test knowledge score of old age people regarding control and prevention of cataract

The frequency and percentage distribution of senior people's pre-test knowledge scores on cataract control and prevention are displayed in Table 4 and Figure 2. In contrast, 54% of them had adequate knowledge, 46% had moderately adequate knowledge, and 2% had adequate knowledge after taking the post-test. Of them, 46% had inadequate knowledge, 52% had a moderately adequate level of knowledge, and 2% had an adequate level of knowledge. More successful was the self-instructional programme on cataract care and prevention.

## Section C: Assess the effectiveness of knowledge on control and prevention of cataract among old age peoples

Table 5 shows that the paired 't' test value was calculated to analyze the effectiveness between pre and post-test knowledge scores of old age peoples. The paired 't' value for knowledge was 18, which is high when compared with table value 2 at 49 degrees of freedom. The self-instructional module of knowledge on control and prevention of cataract among old age peoples was more effective.

Table 6 shows that the pre-test knowledge score of mean was 9.16, standard deviation was 3, mean percentage was 30.53%, whereas post-test knowledge score of mean was 14.5, standard deviation was 2.16, mean percentage was 48.33%. The result revealed that the self-instructional module on control and prevention of cataract had significant effect in increasing knowledge of old age peoples.

## Section D: Association between pre-test knowledge scores of old age peoples with their demographic variables:

Table 7 shows that the chi square was calculated to find out the association between pre-test knowledge scores of old age people with their selected demographic variables regarding control and prevention of cataract. It is revealed that there was no significant association found between pre-test knowledge scores and other demographic variables.

## Discussion:

The purpose of this study was to evaluate the efficacy of a self-instructional module on cataract control and prevention among senior citizens living in the Namakkal District's Olappalayam rural. Cataracts continue to be a deadly ailment in rural Namakkal, just as they are in neighbouring districts. According to previous study, 60% of the participants who were 65 years of age or older had a cataract diagnosis [22]. Nonetheless, our research revealed that women were far more likely than men to get cataracts. According to a study conducted in a rural population in Cuddalore, there was a sharp increase (64.9%) in the number of cataract cases in those over 60 years of age [23]. The prevalence of cataracts in this study was also found to be significantly higher with advancing age in females [24]. Because there is a dearth of research on the gender-specific prevalence of estrogen deficiency in cataract development, the prevalence of cataract was primarily focused on a rural female population aged 60 and above [25] Nonetheless, a few of studies have found that women are substantially more likely than men to develop cataracts [26].

The first objective was to assess the pre-test level of knowledge regarding prevention of cataracts among old age people. In this study, a standardized questionnaire was used to gauge the pre-test level of knowledge of cataracts. Pre-test knowledge levels were found to be inadequate in 23 (46%) of the participants, intermediate in 26 (52%) of the participants, and adequate in 1 (2%) of the participants.

The aforementioned study backs up the findings of a cross-sectional study conducted by Kaur A & Elango (2014) to gauge the level of cataract knowledge among 60 adults in a chosen hospital in Punjab's Sangrur region [27]. An organised knowledge questionnaire was used to evaluate the knowledge, and an attitude assessment was performed. The results of the study showed that knowledge and attitude levels after the intervention were considerably greater than those before it. Thus, the researcher came to the conclusion that the most effective way to prevent cataracts and postpone their emergence in high-risk individuals is to educate the community about the self-instructional module [28].

The second objective was to evaluate the effectiveness of self-instructional module on knowledge regarding prevention of cataract among old age people.

The current research Only 23 (46%) of the cataract patients had moderate knowledge on the post-test, compared to the majority (54%) who had acquired enough knowledge after completing the self-instructional module. Furthermore, the knowledge post-test mean score of 14.5 was higher than the knowledge pre-test mean score of 9.16. At the 0.05 level of significance, the derived 't' value (17.67) was more than the table value, indicating a significant difference in the pre-test and post-test levels of knowledge about cataract prevention among the elderly. As the aforementioned result illustrates, the self-instructional module greatly benefited the senior population's acquisition of knowledge about cataract prevention.

## Conclusion:

According to the study's findings, older adults knew less about cataract prevention in the pre-test than they did in the post-test, but more of them knew enough about it. The self-instructional module on control and prevention of cataracts appears to have had an impact as the mean post-test knowledge scores exceeded the mean pre-test knowledge scores. There are restrictions on generalization because the research study's constraints were restricted to Nammakal District, a specific geographic location. The current study included a sample size restriction of 50 participants and a four-week study period.

## Recommendations:

The study's broad scope allows for the generalization of the results, and similar studies can be conducted in hospitals and other institutions by the student nurses to gauge their understanding of how to prevent cataracts in older adults.

## Funding sources:

No funding was received for this research.

## Figures and Tables

**Table 1:** Research design

**Pre test**	**Intervention**	**Post test**
1	X	2
01 - Pretest to assess the level of knowledge regarding control and prevention of cataract among old age peoples.	X - Intervention (Self Instructional Module)	02 - Post-test to assess the effectiveness of the self-instructional module on control and prevention of cataract among old age peoples.

**Table 2:** Scoring procedure for knowledge level

**Category**	**Range of scores**
Inadequate	Below 50%
Moderately adequate	51-70%
Adequate	Above 70%

**Table 3:** Reveals that the demographical variables of old age peoples

**S.NO.**	**Demographic variables**	**No of old age peoples**	
		**Frequency(F)**	**Percentage(%)**
1	**Age**
	a.55-65	15	30
	b.65-75	30	60
	c.above75	5	10
2	**Gender**
	a.Male	19	38
	b.Female	31	62
3	**Religion**
	a.Hindu	42	84
	b.Christian	7	14
	c.Muslim	1	2
4	**Marital status**
	a.Married	44	88
	b.Unmarried	7	14
	c.Widow	1	2
5	**Education**
	a.No formal education	28	56
	b.Primary school	18	36
	c.High school/Higher secondary	4	8
	d.Graduate	-	-
6	**Occupation**
	a.Government job	10	20
	c.private job	18	36
	d.Housemade	22	44
7	**Income**
	Rs.5000-10,000	31	62
	Rs.10,000-15,000	15	30
	Rs.15000	4	8
8	**Type of family**
	a.Nuclear family	28	56
	b.Joint family	14	28
	c.Extented family	8	16
9	**Food habits**
	a.Vegetarian	16	32
	b.Non-vegetarian	34	68
10	**Place of treatment**
	a.Health center	20	40
	b.Government Hospital	11	22
	c.Private Hospital	19	38

**Table 4:** Frequency and percentage distribution of pre and post-test knowledge scores of old age peoples regarding control and prevention of cataract

**S.NO.**	**Level of knowledge**	**Range of marks**	**Pre Test**		**Post Test**	
			**F**	**%**	**F**	**%**
1	Inadequate	0-10	23	46	0	0
2	Moderately adequate	10-20	26	52	23	46
3	Adequate	21-30	1	2	27	54
4	Total		50	100	50	100
						N=50

**Table 5:** 't'test value and 'p'value to analyze the effectiveness between pre and post-test knowledge scores

**Level**	**'t'value**	**Df**	**Table value**	**'p'value**	**Inference**
Knowledge	17.67	49	2	P<0.05	Significant
					N=50

**Table 6:** Mean, standard deviation, mean percentage and in mean percentage of pre and post-test knowledge of old age peoples

**Level**	**Maximum scores**	**Level of knowledge**			
		**Mean**	**SD**	**Mean%**	**Difference in mean (%)**
Knowledge	30				
Pre test		9.16	3	30.53%	
Post test		14.5	2.16	48.33%	17.80%

**Table 7:** Chi sqaure value of association between pre-test knowledge scores of peoples with their demographic variables

	**Demographic**	**Level of knowledge**				
**S.No.**	**Variables**	**X2 value**	**Df**	**Table value**	**P value**	**Inference**
1	Age	5.39	4	9.49	p>0.05	NS
2	Sex	0.83	2	5.99	P>0.05	NS
3	Religion	0.5	4	9.49	p>0.05	NS
4	Marital status	0.76	6	12.59	p>0.05	NS
5	Education	0.77	4	9.49	p>0.05	NS
6	Occupation	2.38	4	9.49	p>0.05	NS
7	Income	0.23	4	9.49	p>0.05	NS
8	Types of family	1	4	9.49	p>0.05	NS
9	Food habits	0.03	2	5.99	P<0.05	S
10	Place of treatment	0.34	4	9.49	P>0.05	NS
P=0.05
PS=Significant
PNS=Non-significant
P(N=50)

## References

[1] https://pubmed.ncbi.nlm.nih.gov/30969521/.

[2] https://ieeexplore.ieee.org/document/8642133.

[3] Fikrie A (2021). BMC Ophthalmol..

[4] Vashist P (2022). PLoS One..

[5] https://apps.who.int/gb/ebwha/pdf_files/WHA66/A66_11-en.pdf.

[6] Mencucci R (2023). Front Med (Lausanne)..

[7] Khan J (2023). Scientific Reports..

[8] Chi Q (2022). Annals of palliative medicine..

[9] Mohammad NS (2022). Pakistan Journal of Medical Sciences.

[10] Rui F (2022). BMC public health..

[11] Cook C, Qureshi B (2005). Community Eye Health..

[12] Xiao-wei T (2011). Chinese J ophthalmol..

[13] Salunke S (201). Indian J public health..

[14] Abrol S (2021). Community Eye Health..

[15] Khanna RC (2020). Indian J Ophthalmol..

[16] Rao G.N. (2015). Eye (London, England)..

[17] Tan  Xuhua (2020). Ann Transl Med..

[18] Shu Yiyang (2023). Clin Epidemiol..

[19] Cicinelli MV (2020). Indian J Ophthalmol..

[20] https://pubmed.ncbi.nlm.nih.gov/33275950/.

[21] Deshpande M. (2008). Med J Armed Forces India..

[22] Singh S (2019). India. Indian J Ophthalmol..

[23] Doss M.S.J. (2021). Int J Community Med Public Health..

[24] Fang R (2022). BMC Public Health..

[25] Prasad M (2020). The British journal of ophthalmology.

[26] Madeleine Z. (2016). Maturitas..

[27] Amarveer K (2022). International Journal of Geriatric Nursing..

[28] Sankar B (2022). Iranian journal of nursing and midwifery research..

